# Impulse Noise: Can Hitting a Softball Harm Your Hearing?

**DOI:** 10.1155/2014/702723

**Published:** 2014-03-20

**Authors:** Korrine Cook, Samuel R. Atcherson

**Affiliations:** Department of Audiology and Speech Pathology, University of Arkansas for Medical Sciences, University of Arkansas at Little Rock, 2801 South University Avenue, Little Rock, AR 72204, USA

## Abstract

The purpose of this study is to identify whether or not different materials of softball bats (wooden, aluminum, and composite) are a potential risk harm to hearing when batting players strike a 12′′ core .40 softball during slow, underhand pitch typical of recreational games. Peak sound pressure level measurements and spectral analyses were conducted for three controlled softball pitches to a batting participant using each of the different bat materials in an unused outdoor playing field with regulation distances between the pitcher's mound and batter's box. The results revealed that highest recorded peak sound pressure level was recorded from the aluminum (124.6 dBC) bat followed by the composite (121.2 dBC) and wooden (120.0 dBC) bats. Spectral analysis revealed composite and wooden bats with similar broadly distributed amplitude-frequency response. The aluminum bat also produced a broadly distributed amplitude-frequency response, but there were also two very distinct peaks at around 1700 Hz and 2260 Hz above the noise floor that produced its ringing (or ping) sound after being struck. Impulse (transient) sounds less than 140 dBC may permit multiple exposures, and softball bats used in a recreational slow pitch may pose little to no risk to hearing.

## 1. Introduction

Dangerous noise levels in a sporting event such as football (soccer), basketball, or baseball could come from crowd noise, referee whistles, and sporting equipment. Stadium or indoor area employees who work routinely in these environments can also be at risk of loud noise exposure [1–3]. National Football League (NFL) games have been measured to range between 91 and 95 dBA [2], which can have an impact on all individuals involved. In indoor hockey arenas, collegiate games can reach levels from 81 to 96 dBA, while semiprofessional games can reach levels from 85 to 97 dBA. A study involving two spectators wearing personal noise dosimeters measured at three different 2006 Stanley Cup Final games recorded levels between 100 and 104 dBA [4]. In addition, audiometric testing revealed temporary threshold shifts of 5 to 10 dB on average, but in one participant there was a 20 dB shift.

During any sporting event, fans can increase noise levels by screaming, banging on the seats or bleachers, and, where permitted, using devices such as thundersticks and vuvuzelas. Vuvuzelas are trumpet-like instruments capable of producing sound pressure levels between 125 and 130 dB and those who blow these instruments can have significant distortion-product otoacoustic emission reductions that may lead to hearing loss [5]. Fans who blew the vuvuzelas had the greatest exposure followed by nearby fans less than 1 meter from the vuvuzela. Realistically, in any game where vuvuzelas are permitted, there are probably hundreds of fans using these devices putting many individuals at risk of hearing loss.

Sports officials (referees) who use whistles may be contributing to their own hearing loss and other auditory symptoms such as tinnitus [6]. Moreover, a single whistle blown by experienced officials was reportedly as high as 116 dBA and the 100% noise exposure dose over repeated blows can be reached in as little as 5 sec. Fortunately, whistle blows may not have the same effect on the players or fans unless they are close to the sports official.

Of recent interest and relevance to the present study, a study of modern golf drivers was conducted to determine peak levels and potential risk for hearing loss [7]. This particular study was motivated by 55-year-old right-handed male patient who visited an ear, nose, and throat clinic with complaints of tinnitus and reduced hearing in the right ear. An audiogram revealed a high frequency hearing loss in both ears, but the right ear had a noise-induced hearing loss configuration that was up to 20 dB HL worse than the left ear at 4 and 6 kHz. He reported that he had been playing golf three times a week with a King Cobra LD titanium club and owned the golf driver for about 18 months. Magnetic resonance imaging (MRI) was negative for tumor growths on the auditory nerves. Other than playing golf, the patient reported that he had no significant exposure to occupational or recreational noise. Thus, the investigators designed a study to compare peak sound pressure levels produced by six different thick-faced stainless steel golf drivers with six different thin-faced titanium golf drivers. Results of this study showed that all of the thin-faced titanium drivers produced more intense sound pressure levels than the stainless steel drivers on the order of about 10 dB. The thin-faced drivers produced levels between 120 and 130 dB; however, whether the measurements were A- or C-weighting was not reported. These levels potentially put the individual player and nearby golfing partners at risk of temporary or permanent threshold shift.

The foregoing discussion of sport-related noise exposure from crowds, officiating equipment, or sporting equipment is pervasive. However, we were not aware of studies formally evaluating peak sound pressure levels of softball bats. It is generally well known that aluminum bats produce a characteristic “ping” sound, while wooden bats produce more of a “crack” sound. Often times, the “ping” is perceived much louder, dampens less quickly, and is heard at further distances. For these reasons, the present study was designed to measure peak sound levels of three different softball bat materials (wood, composite, and aluminum) with balls thrown using a recreational slow pitch. This study bears relevance to audiologists and otologists who may encounter patients with noise-induced hearing loss or other auditory symptoms (e.g., tinnitus) due to sporting equipment. When hearing is unprotected from high levels of noise, whether the noise has a continuous or impulse quality, individuals may present with hearing loss (or other auditory processing problems [8]), tinnitus, and a reduced quality of life [4]. Unlike all other hearing loss etiologies, hearing loss caused by noise from occupational or recreational activities is 100% preventable [9]. Noise-induced hearing loss occurs gradually that many people do not discover the adverse effects of noise until it is too late for reversal.

## 2. Methods

### 2.1. Participants

 In this study, a batter participant was tasked to hit softballs using three different softball bats delivered by a pitcher participant, each with amateur and collegiate softball experience. The same two participants were available on two measurement days. All procedures received prior approval by the Human Subjects Review Board at the University of Arkansas at Little Rock (Protocol number 13-058).

### 2.2. Materials and Setting

Each of the three softball bats had a weight of 26.5 ounces and length of 32 inches. All were manufactured by Worth (St. Louis, MO, USA). The specific models used were Storm (aluminum), Mayhem (composite), and Mayhem Ash (wood). Unlike wood and aluminum bats, composite bats are the latest technology and can be made out of graphite-fiber composite or have an aluminum core with a graphite lining. Core .40 softballs were used in this study. A core .40 softball is considered a low core and does not have as much “bounce” as a core .44 or .47. All measurements were performed at an empty community softball complex. Sound measures were only taken on days when the temperature was above 65°F (18.3°C) as temperatures is less than 60°F (15.5°C) degrees can result in damage to the bats.

### 2.3. Instrumentation and Procedures

A sound level meter with oscilloscope setup was used to capture time domain waveforms and also record sound level measurements. The setup included a PC-based laptop with PicoScope software and USB-based PicoScope oscilloscope (Tyler, TX, USA) plugged into the laptop and output of the Brüel & Kjær Type 2250 (Skodsborgvej, Denmark) sound level meter coupled to the PicoScope. A Type 4189 1/2′′ microphone was used to capture the levels of the bat striking the softball. The sound level meter was calibrated before all sound measurements, and all recorded peak levels were measured using a C-weighted dB filter. Although the A-weighted measurements are most commonly reported, C-weighted peak sound levels of 140 dB for impulse-type noise are also often reported as the level that should not be exceeded by some countries and independent organizations [10]. The parameters of the PicoScope were set as follows: channel: A, collection time: 500 ms/div, horizontal zoom: ×1, number of samples: 1 MS, input range: ±2 to 5 V, resolution: 8 bits, and coupling: AC or DC. Following time waveform capture using the oscilloscope, the measurement with the highest peak level was converted to a readable WAV file using MATLAB software (Natick, MA, USA) to perform spectral analysis using Adobe Audition 2.0 software (San Jose, CA, USA). The time duration of each of these measurements was also recorded.

The sound level meter was placed in the opposite batter's box at a spatial position as close to the height and distance of the batter's left ear as possible. To obtain reasonable position, the batter was asked to swing the bat while the height and distance of the microphone were relative to the home plate. This microphone positioning should give the most accurate representation of the effects of the impulse noise of a bat hitting a ball on an individual's hearing, minus any head-torso baffle effect. Slow underhand pitches were delivered from the pitcher's mound at regulation distance for softball, which is 45 feet (13.7 meters). Three controlled pitches were delivered for each material of bat. An average for each scenario was taken after 3 pitches for each bat on each day. An overall average was later taken for each bat type over the two days of measurements.

## 3. Results

**Figure 1:**
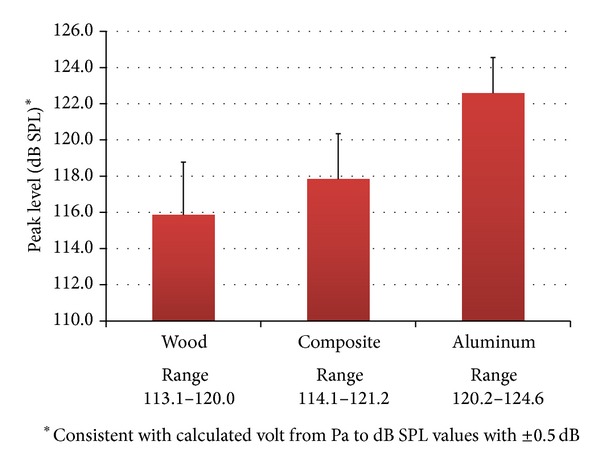
Mean peak levels with standard deviations are shown for each of the three softball bat materials. The range of raw peak levels is also shown. *Peak levels are C-weighted measures.

The peak levels ranged as follows: wood = 113.1–120.0 dBC (*M* = 115.9), composite = 114.1–121.2 dBC (*M* = 117.8), and aluminum = 120.2–124.6 dBC (*M* = 122.6) (see Figure 1). As the results show, the highest peak level recorded was from the aluminum bat. The means of the peak levels from day 1 of each material are as follows: wood = 113.7 dB SPL, composite = 117.0 dBC, and aluminum = 122.8 dBC. The means of the peak levels from day 2 of each material are as follows: wood = 118.0 dBC, composite 118.6 dBC, and aluminum = 122.3 dBC.

**Figure 2:**
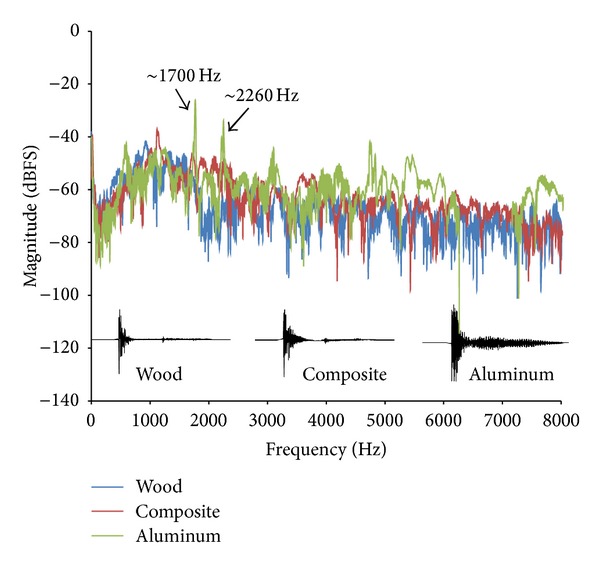
Time domain wave forms and spectra. The time domain wave forms are shown in black with energy lasting on average around 0.1 seconds. As can be seen, the aluminum bat wave form shows signs of “ringing” beyond the initial impulse. The spectra of the three highest bat measurements are shown in blue (wood), red (composite), and green (aluminum). Although all three bat spectra are broad in nature, the aluminum bat has somewhat higher energy between 4000 and 8000 Hz, and the two distinct peaks at approximately 1700 and 2260 Hz are shown. These peaks are at least 20 dB above the rest of the broadband energy (noise floor).

All impulse sounds were no more than 0.111 ms in duration and were submitted to spectral analysis. The composite and wooden bats had a smooth, broad spread of energy and were similar to one another. On the other hand, the aluminum bat produced a spectrum, also broad, but there were clear areas of multiple peaks of energy above the noise floor. Two very distinct peaks emerged from the noise floor around 1700 Hz and 2260 Hz, which coincides with the “ping” of the aluminum bat. Representative time domain waveforms as well as the spectrum for the highest measured sound levels for each bat are shown in Figure 2.

## 4. Discussion

The present study investigated the peak sound pressure levels, time duration, and spectra of three different softball bat materials striking a .40 core softball for potential threat to human hearing. None of the levels recorded met or exceeded the 140 dBC ceiling limit for allowable exposure [10]. However, the high peak impulse levels of all the three bat materials could be a potential hazard for a temporary threshold shift, permanent threshold shift, or other related symptoms if this level is met with repeated exposure in a single game with multiple at-bat opportunities or during a batting practice scenario. The aluminum bats hold the highest risk of causing a temporary threshold shift, permanent threshold shift, or other related symptoms. This situation is not unlike golfing with thin-faced titanium drivers capable of producing peak sound pressure levels of 120 to 130 dB [7]. The “ping” sound produced by the aluminum bat is of the greatest suspect. Spectral analysis with the aluminum bat revealed two significant peaks around 1700 Hz and 2260 Hz, which are comparable to baseball bat data reported by Russell [11]. He showed a comparison between wooden and aluminum bats and found a very similar distinctive “ping” of the aluminum bat producing spectral peaks around 2200 and 2800 Hz. Russell's data as well as the data reported in this present study suggest that aluminum bats share a common characteristic producing spectral peaks that emerge between 1500 and 3000 Hz due to a ringing or “trampoline effect” of the bat. Moreover, these spectral peaks can rise 15 dB or more above the noise floor and could target specific cochlear regions. In a given game with few at-bat chances, the risk to hearing is low. However, seasonal batting practice in an enclosed, reverberant room or golf driver practice at a driving range may well resemble firing guns at a shooting range.

Batting practice facilities are known to have a high reverberant acoustical quality. They usually have concrete flooring and are in sheet metal or exposed concrete block buildings. This environment can become very loud over time and increasingly louder with larger groups of batters hitting during the same practice session. In a practice type setting, a typical batter could easily hit 100 to 150 balls in one session. Moreover, a single batting session may take about 1 or 2 hours and maximum allowable doses may be reached faster than expected.

The present study can only be generalized to recreational softball with slow underhand pitch. It is assumed that peak sound pressure levels are higher with faster underhand softball pitches and faster overhand baseball pitches. While we used a live pitcher with a controlled slow, underhand pitch, future research on other pitch speeds could be explored as well as the use of a pitching machine. In summary, slow underhand softball pitches most likely pose little to no risk to hearing, but batting practice with multiple impulse sound exposures could put an individual at risk of temporary to permanent threshold shift with bat materials that produce high intensity sounds. Hearing protection during long batting practice sessions may be recommended.
